# Prohibitin: a prime candidate for a pleiotropic effector that mediates sex differences in obesity, insulin resistance, and metabolic dysregulation

**DOI:** 10.1186/s13293-019-0239-5

**Published:** 2019-05-22

**Authors:** Yang Xin Zi Xu, Geetika Bassi, Suresh Mishra

**Affiliations:** 1 Department of Physiology and Pathophysiology, College of Medicine, Rady Faculty of Health Sciences, University of Manitoba, Rm. 843 JBRC/715 McDermot Avenue, Winnipeg, MB R3E 3P4 Canada; 2 Department of Internal Medicine, College of Medicine, Rady Faculty of Health Sciences, University of Manitoba, Winnipeg, Canada

**Keywords:** Mitochondria, Sex steroids, *O*-GlcNAc transferase, Estrogen receptors, Androgen receptors, Transgenic mice

## Abstract

Adipocytes and macrophages, the two major constituents of adipose tissue, exhibit sex differences and work in synergy in adipose tissue physiology and pathophysiology, including obesity-linked insulin resistance and metabolic dysregulation. Sex steroid hormones play a major role in sex differences in adipose tissue biology. However, our knowledge of the molecules that mediate these effects in adipose tissue remains limited. Consequently, it remains unclear whether these effector molecules in different adipose and immune cell types are distinct or if there are also pleiotropic effectors. Recently, a protein named prohibitin (PHB) with cell compartment- and tissue-specific functions has been found to play a role in sex differences in adipose and immune functions. Transgenic (Tg) mouse models overexpressing PHB (PHB-Tg) and a phospho-mutant PHB (mPHB-Tg) from the fatty acid binding protein-4 (*Fabp-4*) gene promoter display sex-neutral obesity; however, obesity-related insulin resistance and metabolic dysregulation are male-specific. Intriguingly, with aging, the male PHB-Tg mice developed hepatic steatosis and subsequently liver tumors whereas the male mPHB-Tg mice developed lymph node tumors and splenomegaly. Unlike the male transgenic mice, the female PHB-Tg and mPHB-Tg mice remain protected from obesity-related metabolic dysregulation and tumor development. In conclusion, the sex-dimorphic metabolic and immune phenotypes of PHB-Tg and mPHB-Tg mice have revealed PHB as a pleiotropic effector of sex differences in adipose and immune functions. In this mini-review, we will discuss the pleiotropic attributes of PHB and potential mechanisms that may have contributed to the sex-dimorphic metabolic phenotypes in PHB-Tg and mPHB-Tg mice, which warrant future research. We propose that PHB is a prime candidate for a pleiotropic mediator of sex differences in adipose and immune functions in both physiology and pathophysiology, including obesity, insulin resistance, and metabolic dysregulation.

## Background

Obesity (and its associated metabolic abnormalities) is an increasingly common ailment worldwide. It is a risk factor for a variety of diseases, including metabolic syndrome, type 2 diabetes, cardiovascular disease, and some types of cancer [1–3], which are also growing in parallel with obesity. As the magnitude of obesity-related health problems is enormous, it has been an extensively investigated area of biomedical research for the last four decades. Accumulating evidence over the years has provided substantial proof that adipose tissue-specific immune dysregulation in the form of low-grade inflammation is an integral component of obesity-related metabolic abnormalities [4–6]. Notably, adipose and immune functions display sex differences in physiology and pathophysiology, including adipose tissue distribution and functions, obesity, insulin resistance, and metabolic syndrome [7–10]. In spite of extensive research on adipose tissue biology pertinent to obesity, a number of fundamental questions remain unanswered, especially in the context of sex differences in adipose tissue biology and pathobiology. There are a number of potential factors that may contribute to sex differences in adipose tissue functions in health and disease, including sex chromosomes, sex hormones, mitochondria, and epigenetic factors [11]. However, our knowledge of effector molecules that mediate sex differences in adipose and immune functions remains poor. It is also unclear whether these effector molecules in different adipose and immune cell types are distinct, or if there are common effector molecules, and whether the sex differences in adipose and immune functions influence each other.

Prohibitin (PHB) is an evolutionarily conserved pleiotropic protein, which localizes to different cellular compartments, including the nucleus, mitochondria, and cell membrane. It has cell compartment and cell/tissue-specific functions (reviewed in [12, 13]). For example, PHB has a role in adipocyte differentiation and in cell signaling in various cell types, including different types of immune cells [14–19]. The adipocyte-specific role of PHB appears to be mediated through its mitochondrial functions, whereas immune cell-specific role of PHB appears to be primarily involving membrane-associated cell signaling functions [15–17, 19]. In addition, PHB has a multifaceted relationship with sex steroid hormones, estrogens and androgens, and their receptors (reviewed in [13]). On the one hand, PHB functions as a co-repressor of sex steroid receptors, while on the other, it has been identified as a target gene under sex hormone regulation [20–23]. Most work on PHB and its association with sex steroids and their receptors have been reported in reproductive tissues such as the endometrium, mammary gland, and prostate tissues or their derivative cell lines [20–23]. However, the potential role PHB may play in mediating sex differences in non-reproductive tissues has not been investigated, despite substantial influence of sex steroids on whole body metabolism and in major metabolic tissues, including the adipose tissue, skeletal muscle, and liver tissue.

Recently, we have developed and characterized two transgenic (Tg) obese mouse models that overexpress PHB (PHB-Tg) or a phospho-mutant PHB lacking the tyrsone-114 phosphorylation site (mPHB-Tg) from the fatty acid binding protein-4 (*Fabp-4*) gene promoter (reviewed in [12, 13]). The *Fabp-4* gene promoter was used for simultaneous expression of PHB or mPHB in both adipocytes and in monocytic macrophages/dendritic cells [24–26]. PHB transgenic mice developed obesity independent of diet due to mitochondrial biogenesis in adipocytes [27]. The obese and metabolic phenotypes of PHB-Tg and mPHB-Tg mice have been reviewed recently [12, 13] and therefore will not be discussed here in detail. However, to put things into a perspective, we will briefly point out sex differences in metabolic phenotype of the PHB-Tg and mPHB-Tg mice wherever necessary. In brief, phenotypic characterizations of the PHB-Tg and mPHB-Tg mice have revealed that PHB plays an important role in sex differences in adipose and immune functions [27, 28]. The sex dimorphic phenotypes in PHB-Tg and mPHB-Tg mice include male-specific obesity-related insulin resistance, low-grade chronic inflammation, and obesity-related tumors [27–29], which appear to be a consequence of the cell compartment- and cell type-specific functions of PHB. This finding provided proof that the interplay between PHB and sex steroids is not only limited to reproductive tissues, but also occurs in metabolic tissues and immune cells. This finding may have implications in human health and disease. In addition to sex steroids, PHB interacts with *O*-GlcNAc transferase (OGT; uridine diphospho-*N*-acetylglucosamine:polypeptide β-*N*-acetylglucosaminyl transferase), an X-linked gene recently identified as a mediator for sex differences in a variety of cell types [30–32]. The role of OGT in mediating sex differences involves its cell signaling and transcriptional functions. Interestingly, PHB and OGT share many features related to their regulation and pleiotropic functions (Table 1). These evidences are suggestive of PHB as a candidate pleiotropic effector molecule in producing sex differences in adipose and immune functions.

**Table 1:** The common and unique features of PHB and OGT, which may have a role in mediating sex differences in insulin resistance and metabolic dysregulation

• PHB and OGT interact with each other and are regulated by sex steroids. In addition, PHB functions a co-repressor of sex steroid receptors • PHB and OGT share similar tyrosine motifs, undergo tyrosine phosphorylation in response to insulin, and negatively regulate insulin signaling • Both interact with a number of insulin signaling intermediates • Both localize to mitochondria and have an important role in mitochondrial functions • Both function as transcriptional regulators • In addition, OGT is an X-linked gene, subject to gene dose compensation, and invariably escape from X chromosome inactivation in different cell types	

In this mini-review, we will focus on the various attributes of PHB and the putative mechanisms that may be involved in the role of PHB in sex differences in adipose and immune functions and identify future research direction in this field. Particularly, we will discuss the known relationship of PHB with sex steroid hormones and their mitochondrial attributes. In addition, we will discuss the common features that are shared between PHB and OGT, which may have implications in mediating sex differences in insulin resistance and metabolic dysregulation. Unraveling why and how PHB overexpression in adipocytes and macrophages/dendritic cells confers resistance to obesity-related metabolic dysregulation in the females, but opposite in males, may open innovative sex-specific interventions for obesity, insulin resistance, and metabolic syndrome.

## Prohibitin and sex steroids—a multifaceted relationship

Sex chromosomes, and by extension sex steroid hormones, play a fundamental role in sex differences in the structure, regulation, and function of different cell/tissue types in the body, [33, 34]. However, the various effector molecules that mediate or modulate the effects of sex steroids on sex differences in different tissue types are largely unknown. Emerging evidence suggests that PHB is a pleiotropic effector of sex differences in adipocytes and in monocytic macrophages/dendritic cells and has a relationship with sex steroids [27–29, 35]. The first evidence of the association between PHB family proteins and sex steroids came from the discovery of the repressor of estrogen action (REA) as a co-repressor of estrogen receptors (ERs) [36]. Gene and protein sequence analyses revealed that REA is a homologous protein of PHB that share > 50% sequence homology [36, 37]. As a result, REA was given the name PHB2 (with PHB then being referred to as PHB1). Subsequent studies showed that similar to PHB2, PHB also has the ER co-repressor function and was identified as a target gene for estrogen and ERs [20, 22]. It appears that prohibitins (PHBs) and ERs form a regulatory loop to control each other’s functions and to maintain tissue homeostasis [22]. Subsequently, PHB was also found to function as a co-repressor of androgen receptors (ARs) [38] and was identified as a target gene for androgen and ARs [23]. Interestingly, ERs positively regulate PHB whereas ARs negatively regulate PHB [22, 23], which may have important implications in mediating sex differences. Most findings that showed a multifaceted relationship between sex steroids and PHBs came from reproductive tissues or their derivative cell lines [20–23, 38]. Until recently, it was not known whether this relationship between PHBs and sex steroids exist in other cell or tissue types.

During phenotypic characterization of the PHB-Tg obese mouse model, we found that both male and female PHB-Tg mice developed obesity. However, only male PHB-Tg mice displayed obesity-related metabolic dysregulation, such as impaired glucose homeostasis, insulin sensitivity, and hyperinsulinemia [27]. This would imply that the functional consequences of PHB-induced obesity in male and female PHB-Tg mice are different, suggesting a potential interplay between PHB and sex steroids in adipocytes. To the best of our knowledge, this observation is the first indication of a sex-dimorphic role of PHB in adipose tissue functions. Consistent with a dysregulated metabolic phenotype, crown-like structures in visceral adipose tissue (a sign of macrophage infiltration) and fatty livers were also found in the male PHB-Tg mice [27]. With aging, fatty liver in the male PHB-Tg mice progressed to non-alcoholic steatohepatitis (NASH), and eventually hepatocellular carcinoma (HCC) around 12 months of age [29]. These changes were not observed in female PHB-Tg mice [29], suggesting that the female sex steroid hormone potentially plays a role. Thus, PHB overexpression in adipocytes further amplified sex differences in obesity-related metabolic dysregulation, where the female mice developed resistance and the male mice became susceptible. On the other hand, the mPHB-Tg mouse model shares the sex-neutral obesity and sex-dimorphic metabolic phenotype of PHB-Tg mice, implying that the loss of the tyrosine-114 phosphorylation site in PHB does not affect its mitochondria-mediated adipogenic functions [28]. Despite these phenotypic similarities, the male mPHB-Tg mice did not develop NASH and HCC but instead developed lymph node tumors and splenomegaly around 6 months of age; this was again not observed in the female mPHB-Tg mice [28]. It appears that PHB-mediated sex differences are not limited to adipose tissue biology but are also present in immune functions. It is possible that pre-existing or co-existing immune conditions play a crucial role in the development and progression of obesity-related metabolic diseases. In the case of male mPHB-Tg mice, the loss of tyrosine-114 phosphorylation site altered the disease course observed in the male PHB-Tg mice. Moreover, tumor development in the male PHB-Tg and mPHB-Tg mice provided a proof that obesity-related metabolic abnormalities, such as hyperinsulinemia, facilitate tumor development, because PHB or mPHB by itself does not lead to tumor development in the female transgenic mice [28].

Ovariectomy in the female mPHB-Tg mice suggested a potential role of ovarian estrogens in protection from obesity-related metabolic dysregulation, as the ovariectomized mPHB-Tg mice developed impaired glucose homeostasis and insulin sensitivity similar to their male counterparts [28]. However, as estrogen replacement therapy in ovariectomized mPHB-Tg mice was not performed [28], a potential role of other ovarian factors may not be ruled out. Moreover, obesity-related metabolic abnormalities after ovariectomy were sufficient to induce tumor development in the female mPHB-Tg mice [28]. Interestingly, ovariectomy in the female mPHB-Tg mice prevented weight gain [28], suggesting that PHB depends on sex steroid hormones for producing obese phenotype. Sex steroids may play a modulatory role in PHB function in adipocytes, which brings an additional complexity to their multifaceted relationship. In this context, it is important to note that the loss of estrogens increases adiposity in both rodents and humans [39, 40], which was not observed in the ovariectomized mPHB-Tg mice [28]. A similar finding was also observed in the body weight of ovariectomized PHB-Tg mice; however, they remained protected from metabolic dysregulation [41]. These findings indicate that ovariectomy-induced metabolic dysregulation in mPHB-Tg mice is a conjoint manifestation of adipose and immune-associated alterations [27, 28]. In the male PHB-Tg mice, orchiectomy stopped PHB-induced weight gain and improved metabolic homeostasis, such as glucose tolerance and insulin sensitivity [41]. This would imply that PHB functions differently in male and female adipose tissue in both the presence and absence of sex steroids. This finding may have important implications in aging-related sex differences in health and diseases, as sex steroid hormone levels change throughout an individual’s life and estrogens and androgens differentially regulate PHB expression levels [22, 23]. In other words, PHB may function differently in males and females during different stages of life. Of note, alteration in PHB levels has been found in the context of aging in various model systems [42–44] and in adipocytes from obese patients in relation to aging [45]. In *Caenorhabditis elegans*, PHB is a context-dependent modulator of aging [42], whereas mild caloric restriction in mice (a model for longevity) upregulates the expression of PHB in livers [45]. PHB may play a similar role in monocytic macrophages and dendritic cells, which may have contributed to the gonadectomy-induced alterations in the obesity-related metabolic phenotype in the PHB-Tg and mPHB-Tg mice. Despite challenges associated with deciphering the relative contributions of adipocytes and macrophages/dendritic cells in phenotypic changes in the PHB-Tg and mPHB-Tg mice, these mouse models have revealed PHB as a pleiotropic effector of sex differences in adipose and immune functions. Our findings raise a number of important questions about sex differences in adipose tissue functions, insulin resistance, and metabolic dysregulation and have opened potential research topics in this field.

## Are the sex-dimorphic effects of PHB in adipose tissue mediated via mitochondria?

PHB (and its homologous protein PHB2) plays a crucial role in mitochondrial biology, where it functions as a chaperone for mitochondrial proteins and phospholipids [46–48]. PHB or PHB2 overexpression enhances mitochondrial functions, whereas its knockdown compromises mitochondrial functions in cell and animal models [16, 27, 49]. In vitro, PHB overexpression in preadipocytes increases adipocyte differentiation, whereas PHB knockdown has the opposite effect on adipocyte differentiation [14, 15]. In vivo, although both male and female PHB-Tg mice developed obesity similarly, obesity-related metabolic dysregulation was observed only in the male [27]. Thus, the sex differences in obesity-related insulin resistance and metabolic dysregulation are further widened in PHB-Tg mice. The PHB-Tg mice developed obesity because of mitochondrial biogenesis in adipocytes [27]. Based on this finding, the role of PHB in sex-dimorphic adipocyte functions may be mediated through the mitochondria. This was evidenced by adipokine analysis to which increased adiponectin and decreased leptin were found in female compared with male PHB-Tg/mPHB-Tg mice [27, 28]. In addition, sex steroids play different roles in mitochondrial biology. For example, estrogens are generally considered to facilitate mitochondrial biogenesis [50], whereas results on testosterone function in mitochondrial biology are controversial [51, 52]. Recently, Bajpai et al. [53] have provided proof that ARs contain mitochondrial localization signal and play a role in mitochondrial function in prostate cancer cells. Previously, a multifaceted relationship between PHB and ARs has been reported in prostate cancer cells in the context of genomic actions of androgens. It would be interesting to know whether the interplay between PHB and androgens in prostate cancer cells also occur in mitochondrial compartment. As sex steroid hormones are intrinsic to sex differences and have a multifaceted relationship with PHB, it is likely that the interplay between PHB and sex steroids in mitochondrial biology plays a role in the sex differences in adipose tissue functions. Differential alterations in the gonadectomy-induced metabolic changes in the male and female PHB-Tg and mPHB-Tg mice, as revealed by glucose and insulin tolerance tests [28, 41], indicate that this is indeed appears to be the case. It is anticipated that further investigation into the adipose tissue and monocytic macrophages/dendritic cells of PHB-Tg and mPHB-Tg mice will provide a clear understanding of the interplay between PHB, sex steroids, and mitochondria in the specific cell type. In this context, it is important to note that mitochondrial dysregulation is a common feature of obesity-related abnormalities in different metabolic tissues [54–56]. In addition to sex steroids, the role of mitochondria in sex differences in obesity-related metabolic abnormalities may involve epigenetic mechanisms. Mitochondrial DNA copy numbers have been reported to play a role in epigenetic changes in the nuclear genome [57]. PHB is known to interact with mitochondrial transcription factor A (Tfam) [58], and PHB-Tg mice have increased mitochondrial DNA copy number in the adipose tissue [27]. Moreover, a number of nuclear-encoded mitochondrial genes located on X chromosomes, are subject to escape from X chromosome inactivation [59, 60], which may further contribute to sex differences in obesity-related insulin resistance and metabolic dysregulation. Thus, a combination of PHB and sex steroid interplay as well as escape from X chromosome inactivation of nuclear-coded mitochondrial gene/proteins may contribute to sex differences in metabolic health and disease.

## OGT and PHB—potential partners in mediating sex differences in insulin resistance and metabolic dysregulation

OGT is a glycosyltransferase that catalyzes the addition of a single GlcNAc molecule in *O*-glycosidic linkage to serine or threonine residues (*O*-GlcNAcylation) in a diverse array of proteins. It is a reversible posttranslational modification at the serine and threonine residues mediated by the GlcNAc cycling enzymes OGT and *O*-GlcNAc amidase (OGA) [61]. *O*-GlcNAcylation often occurs at the site of, or proximal to, the same serine and threonine residues modified by kinases [61], and this competition permits a dynamic interplay that can alter signaling and protein functions [61–63]. Recent evidence suggests that OGT is a pleiotropic effector that mediates sex-dimorphic functions [30–32]. For example, sex differences in placental OGT mediate the effects of prenatal stress on neurodevelopmental programming [30]. Subsequently, canonically repressive epigenetic modification H3K27me3 (tri-methylation of lysine-27 on histone H3 subunit) was identified as one mechanism wherein sex differences in OGT confer variation in vulnerability to prenatal insults via establishing sex-specific trophoblast gene expression patterns [31]. It has been shown that high levels of H3K27me3 in the female placenta create resilience to the altered hypothalamic programming associated with prenatal stress exposure [31]. Notably, the OGT gene is located on the X chromosome in both human and mice [32] and is known to be under the control of dosage compensation mechanisms [64, 65]. In addition, OGT has been identified among genes that invariably escape from X chromosome inactivation (XCI) in different cell or tissue types [66]. However, the gene encoding OGA is not present on the X chromosome. Thus, a differential expression levels, or the ratio of the *O*-GlcNAc cycling enzymes, OGT, and OGA, may exist in male and female tissues, which could contribute to sex differences in cell signaling and transcriptional regulation, as well as mitochondrial functions. This is because in addition to cell signaling functions, OGT is a transcriptional regulator that could regulate diverse expression networks [32] and localizes to mitochondria, where it plays a role in the regulation of mitochondrial structure and functions [67]. In the context of cell signaling, it is important to note that a number of insulin signaling intermediates undergo *O*-GlcNAcylation, which plays a role in insulin resistance [68–71]. For example, the *O*-GlcNAcylation of insulin receptor substrate-1 (IRS1) occurs within or in close proximity of tyrosine phosphorylation sites, which are involved in the interaction between IRS1 and phosphoinositide 3-kinase (PI3K) [68, 69, 71]. The *O*-GlcNAcylation of IRS1 has been shown to interfere with its interaction with PI3K and attenuate insulin signaling downstream of the IRS1 [68, 69, 71]. In addition to *O*-GlcNAcylation, the phosphorylation of IRS1 by an inhibitor of nuclear factor kappa-B kinase subunit beta (IKKβ), protein kinase C (PKC), and c-Jun N-terminal kinase (JNK) at serine residues has been reported to negatively regulate insulin signaling [72]. However, it is not known whether the serine phosphorylation of IRS1 that is involved in inducing insulin resistance is also subjected to *O*-GlcNAcylation. Thus, a possibility exists that the *O*-GlcNAcylation of such residues may prevent their negative effects on insulin signaling and may be differentially regulated in male and female due to escape of OGT from the XCI.

In addition to insulin resistance, mitochondrial dysregulation in metabolic tissues is a well-established feature of obesity-associated insulin resistance and metabolic dysregulation [54–56]. Notably, the *O*-GlcNAcylation of mitochondrial proteins has been implicated in obesity and hyperglycemia-related mitochondrial dysregulation [73, 74]. However, it is not known whether differential *O*-GlcNAcylation of insulin signaling intermediates and mitochondrial proteins play a role in sex differences in insulin resistance and metabolic dysregulation. Recently, OGT-related mitochondrial motility has been shown to be associated with sex differences and exercise effects in depression induced by prenatal exposure to glucocorticoids [75]. Moreover, the *O*-GlcNAcylation of a number of proteins in cardiomyocytes has been reported to have protective effects from ischemia-perfusion injury [76, 77]. Sex differences are known to exist in cardiovascular diseases [78, 79], which is a major complication of obesity and type 2 diabetes. It would be interesting to know if the OGT escape from the XCI in different metabolic tissues, which play a role in sex differences in cellular functions, either directly as a transcriptional regulator, or through the *O*-GlcNAcylation of cell signaling and mitochondrial proteins.

## Does alternate *O*-GlcNAcylation and phosphorylation of PHB at common sites play a role in sex-dimorphic functions?

Previously, we have shown that PHB physically interacts with OGT and is *O*-GlcNAcylated at the serine-121 and threonine-258 residues [80]. Moreover, PHB undergoes tyrosine phosphorylation in response to insulin stimulation, which in turn negatively regulates insulin signaling in multiple cell types [81, 82]. The tyrosine phosphorylation sites (tyrosine-114 and tyrosine-259) and the *O*-GlcNAcylation sites (serine-121 and threonine-258) in PHB are in close proximity, and they regulate each other [80]. The tyrosine phosphorylation of PHB facilitates *O*-GlcNAcylation, whereas *O*-GlcNAcylation has the opposite effect on tyrosine phosphorylation [80]. Interestingly, threonine-258 in PHB is an Akt (protein kinase B) phosphorylation site, which positively regulates insulin signaling through a process that involves its interaction with phosphatidylinositol (3,4,5)-triphosphate [82, 83]. Similarly, the threonine-308 phosphorylation site in Akt, which is required for Akt activation, is also subjected to *O*-GlcNAcylation [70] and potentially contributes to the development of insulin resistance. Thus, a possibility exists that alternate phosphorylation and *O*-GlcNAcylation of insulin signaling intermediates, including PHB, at common sites or adjacent sites may cause sex differences in insulin resistance and consequently metabolic dysregulation. Of note, similar to PHB, OGT also undergoes tyrosine phosphorylation and *O*-GlcNAcylation in response to insulin, and tyrosine phosphorylation enhances *O*-GlcNAc transferase activity, which in turn negatively regulates insulin signaling [71]. Moreover, PHB and OGT are target genes for androgen receptors [23, 84], and both interact with a number of epigenetic and transcriptional regulators [85–91], play a role in the regulation of sex differences in a number of cell or tissue types, and contain similar tyrosine motifs [80]. For example, OGT has been found to complex with ten-eleven translocation proteins-1,2,3 (TET1,2,3) that regulators of DNA demethylation [85, 86], transcriptional repressors SIN3A and histone deacetylases [87], transcriptional activator HCF-1 (host cell factor-1) [88], histone methyltransferase MLL5 (mixed lineage leukemia-5) [89], and modify histone tails [90, 91]. In addition, ERs are known to interact with OGT and undergo *O*-GlcNAcylation (including alternate phosphorylation and *O*-GlcNAcylation at the same residue), which influence their transcriptional activities [92, 93]. Similarly, PHB functions as a transcriptional co-regulator with BRG1 (brahma-related gene-1), HDAC1 (histone deacetylase-1), and p300 (histone acetyltransferase p300), as well as a co-repressor of androgen and estrogen receptors [94, 95]. Although PHB clearly acts as a key transcriptional regulator, how PHB itself is regulated remains largely unknown. Theiss et al. [96] have shown that the *PHB* gene promoter contains functional interleunin-6 (IL-6) response element, and we have found that insulin regulates PHB expression in adipocytes [14]. Thus, PHB expression and functions may be altered in obesity and obesity-related abnormalities, such as insulin resistance and low-grade chronic inflammation. Furthermore, accumulating evidence suggests that PHB and OGT play an important role in different immune cell types [27–32, 97] and OGT is highly expressed in lymphocytes and lymphoid tissues [32, 98, 99]. Thus, PHB and OGT may be a part of sex steroid signaling in metabolic tissues and in the regulation of insulin signaling in a sex-specific manner. PHB and OGT may also be regulated concurrently at the transcriptional and protein levels, which may contribute to sex differences in metabolic and immune functions.

## Does PHB2 have a role in sex differences in obesity, insulin resistance, and metabolic dysregulation?

Some of the pleiotropic attributes of PHB is also shared by its homologous protein PHB2 [12, 18], including a number of phosphorylation sites that have been implicated in their cell signaling functions and intracellular trafficking [100, 101]. For examples, PHBs have been shown to play a crucial role in pancreatic beta cell function [48, 102] and in the maturation of T cells [18]. Despite similarities between PHB and PHB2, their functions do not appear to be redundant or compensated by each other. Rather, the knockdown or deletion of one member often leads to substantial decrease in the protein level of other members [15, 48, 103, 104]. However, to the best of our knowledge, a role of PHB2 in sex differences has not been reported. As PHBs form heterodimers in the inner mitochondrial membrane, play a crucial role in mitochondrial biology, and have multifaceted relationship with sex steroids and their receptors, it is possible that PHB2 has sex-dimorphic functions, which requires further investigation.

## Conclusion

**Fig. 1:**
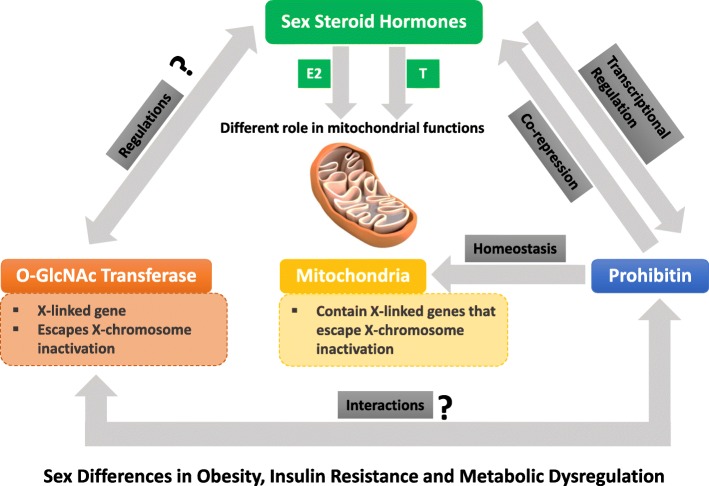
Schematic of potential interplay between sex steroid hormones, prohibitin, *O*-GlcNAc transferase, and mitochondria in the sex difference in obesity, insulin resistance, and metabolic dysregulation. Sex steroid hormones differentially regulate mitochondrial biology; E2 and T regulate prohibitin at the transcription level; and prohibitin in turn acts as co-repressor of sex steroid actions. Prohibitin is essential for maintaining mitochondrial homeostasis. *O*-GlcNac transferase and some of the nuclear-encoded mitochondrial genes are X-linked and can escape X chromosome inactivation. *O*-GlcNac transferase shares similar features as prohibitin and may be subject to regulation by sex steroid hormones. E2 estradiol, T testosterone

Sex differences are fundamental to the biology and pathobiology of human health and disease, and these differences are apparent in adipose and immune functions. However, this basic tenet of human adipose and immune functions has not yet been capitalized for the development of sex-based therapeutics for more effective treatment outcomes. A major hurdle has been our poor knowledge of effector molecules, which mediate these differences in health and disease. The discovery of the pleiotropic attributes of PHB and OGT in mediating sex differences in different cell or tissue types is a step forward in remediating this. There are a number of potential mechanisms, which may work in a cell compartment- and cell type-specific manner; however, a bi-faceted relationship between PHB and sex steroids appears to be central to it (Fig. 1). This unique relationship between PHB and sex steroids may increase the likelihood of targeting PHB for sex-based precision medicine, especially for obesity-related metabolic diseases. In addition to adipocytes and monocytes (macrophage and dendritic cells), PHBs play a role in pancreatic beta cells, lymphocytes, and mast cells [17–19]. We anticipate that decoding the complex relationship between the PHBs and sex steroids in different cellular compartments and cell types will lead to new insights into the underlying mechanisms and sex-specific therapeutic opportunity. Targeting these pathways would help to build a fresh approach that will contribute to innovative regimens for the sex-specific prevention and treatment of obesity, insulin resistance, and metabolic syndromes.

## Abbreviations

Akt: Protein kinase B
AR: Androgen receptors
BRG1: Brahma-related gene-1
E2: Estradiol
ER: Estrogen receptors
*Fabp4*: Fatty acid binding protein-4
H3K27me3: Trimethylation of lysine-27 on histone H3 subunit
HCC: Hepatocellular carcinoma
HCF1: Host cell factor 1
HDAC1: Histone deacetylase 1
IKKβ: Inhibitor of nuclear factor kappa-B kinase subunit beta
IRS1: Insulin receptor substrate 1
JNK: c-Jun N-terminal kinase
MLL5: Mixed lineage leukemia 5
mPHB-Tg: Mutant PHB-Tg mouse model
NASH: Non-alcoholic steatohepatitis
OGA: *O*-GlcNAc amidase
*O*-GlcNAcylation: Protein modification at serine or threonine residue by *O*-linked *N*-acetylglucosamine
OGT: *O*-GlcNAc transferase (uridine diphospho-*N*-acetylglucosamine:polypeptide β-*N*-acetylglucosaminyltransferase)
p300: Histone acetyltransferase p300
PHB: Prohibitin
PHB2: Prohibitin 2
PHBs: Prohibitin (also known as prohibitin-1) and prohibitin-2
PHB-Tg: An obese mouse model developed by prohibitin-induced mitochondrial remodeling in adipocytes and macrophages/dendritic cells
PI3K: Phosphoinositide 3-kinase
PKC: Protein kinase C
REA: Repressor of estrogen activity
SIN3A: SIN3 transcription regulator family member A
T: Testosterone
Tafm: Mitochondrial transcription factor A
TET1, 2, 3: Ten-eleven translocation proteins-1, 2, 3
Tg: Transgenic
XCI: X chromosome inactivation
